# Chicken skin based Milli Watt range biocompatible triboelectric nanogenerator for biomechanical energy harvesting

**DOI:** 10.1038/s41598-023-36817-7

**Published:** 2023-06-22

**Authors:** Muhammad Umair Khan, Eman Mohammad, Yawar Abbas, Moh’d Rezeq, Baker Mohammad

**Affiliations:** 1 Department of Electrical Engineering and Computer Science, Khalifa University, Abu Dhabi, 127788 UAE; 2 System on Chip Lab, Khalifa University, Abu Dhabi, 127788 UAE; 3 Sheikh Khalifa Medical City Abu Dhabi, Abu Dhabi, UAE; 4 Department of Physics, Khalifa University, Abu Dhabi, 127788 UAE

**Keywords:** Energy science and technology, Energy harvesting, Devices for energy harvesting

## Abstract

This work reports a high-performance, low-cost, biocompatible triboelectric nanogenerator (TENG) using chicken skin (CS). The device is suitable to power wearable devices, which is critical to adapt electronics in monitoring, predicting, and treating people. It also supports sustainability by providing a cost-effective way to reduce the poultry industry's waste. It has been shown here that CS-derived biowaste is an effective means of generating tribopositive material for TENGs. The CS contains amino acid functional groups based on (Glycine, Proline, and Hydroxyproline), which are essential to demonstrate the electron-donating ability of collagen. The skin was cut into 3 × 3 cm^2^ and used as the raw material for fabricating the TENG device with a stacking sequence of Al/Kapton/spacing/CS/Al. The chicken skin-based TENG (CS-TENG) is characterized at different frequencies (4–14 HZ) using a damping system. The CS-TENG produces an open-circuit voltage of 123 V, short-circuit current of 20 µA and 0.2 mW/cm^2^ of a power density at 20 MΩ. The biocompatible CS-TENG presents ultra-robust and stable endurance performance with more than 52,000 cycles. The CS-TENG is impressively capable of scavenging energy to light up to 55 commercial light-emitting diodes (LEDs), a calculator, and to measure the physiological motions of the human body. CS-TENG is a step toward sustainable, battery-less devices or augmented energy sources, especially when using traditional power sources, such as in wearable devices, remote locations, or mobile applications is not practical or cost-effective.

## Introduction

**Figure 1:**
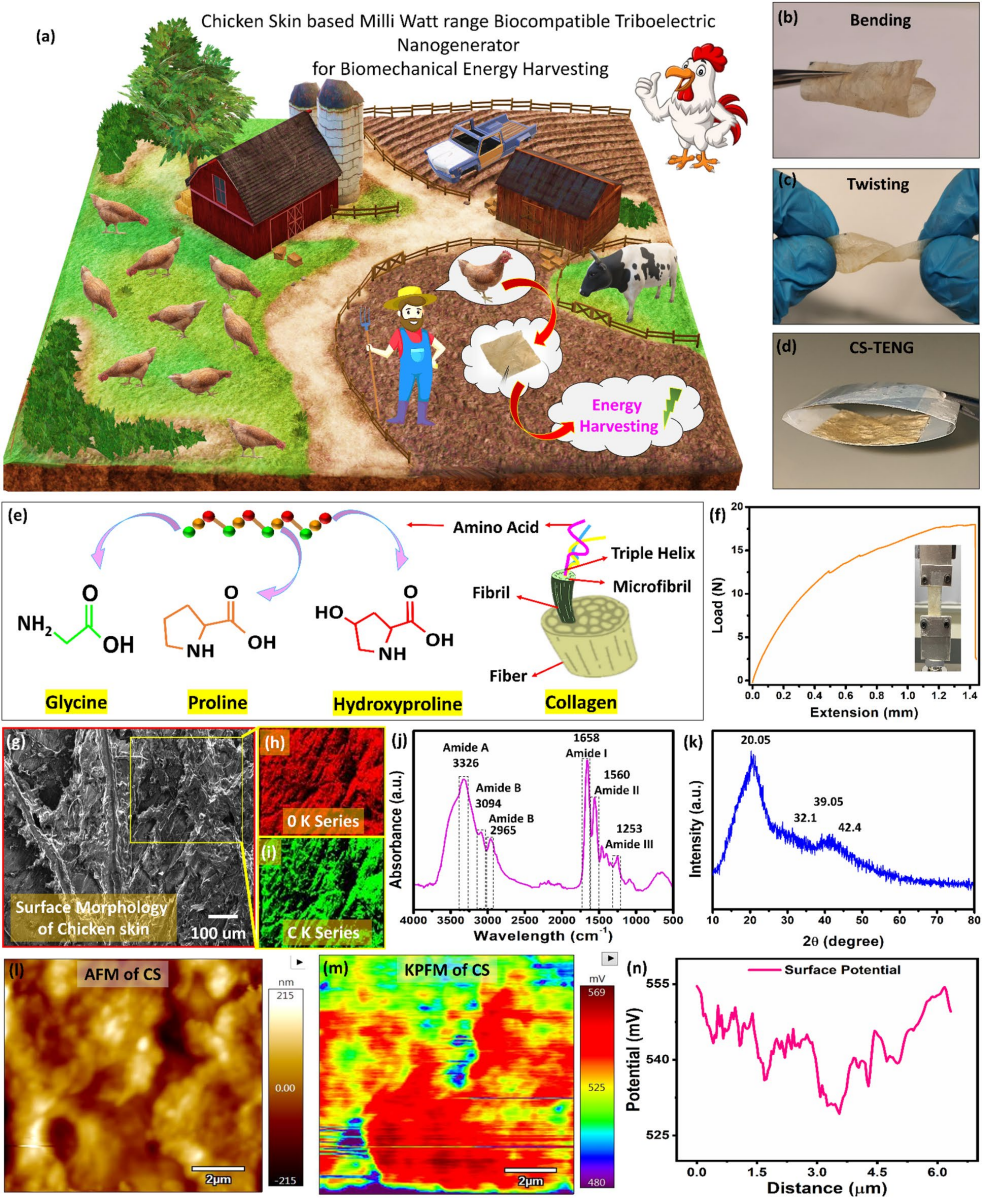
(**a**) Schematic describing the processing of CS waste for energy harvesting. (**b**) Bending and (**c**) twisting of CS-TENG. (**d**) The realized image of CS-TENG with device structure Al/Kapton/CS/Al. (**e**) The structure of collogen and amino acids of CS. (**f**) The load effect of CS to examine the mechanical strength. (**g**) The surface morphology of CS shows the element mapping of (**h**) O-K-series and (**i**) C-K-series. (**j**) The FTIR spectrum of CS. (**k**) The XRD spectrum of CS. (**l**) Topography of CS. (**m**) KPFM results showing the electropositive charge on the CS. (**n**) KPFM potential plot of CS.

With the recent development of the miniaturization technology of portable electronic devices, energy consumption has drastically changed from centralized to decentralized. Recently, mobile and battery-operated electronic devices have become an integral part of almost all aspects of our daily lives, such as wearable health devices to monitor, predict and treat people^1–3^, implantable devices^4^, and portable communication^5,6^. Despite this adaptation, many challenges still need to be overcome, such as security, cost, and energy^7^. To meet these challenges for healthcare, the first one is to provide a safe, reliable, and efficient energy source for these smart wearable electronics^7–10^. However, there are many obvious disadvantages associated with traditional batteries, including their weight, energy density, short charge time, maintenance cost, and incompatibility with the body^11,12^. A self-powered technology has emerged in energy harvesting, enabling it to create energy-autonomous, wearable devices^7,13,14^. The triboelectric nanogenerator (TENG) could be used as a self-powered sensor which converts biomechanical energy into electrical energy^15^. Biomechanical energy refers to the movement of the human body, which is often wasted or neglected in our daily lives^16^. In contrast, using human physiological signals in healthcare and human–computer interaction is very important^17^. The output of the TENG device can be further processed using analogue to digital circuits for human body vibration for sensing and energy harvesting^18^. The TENG is a potential candidate for developing self-powered electronics due to many advantages, including ease of handling, high output performance, high efficiency, and a low-cost fabrication process^19^. TENG's working mechanism is based on a principle of contact electrification that addresses the effects of different electron affinities being created between two materials in contact with one another through an electrostatic induction^15,20^. There is a critical role for electron affinity in producing triboelectric charges caused by electropositive and electronegative materials; therefore, choosing a triboelectric material is a top priority in designing TENGs^21^. Organic and inorganic materials, such as metal oxides, organic, inorganic materials and metal-oxide frameworks, are tribopositive materials^22^ which are nonrenewable, nonbiodegradable and expensive to produce^23–25^. This is why there is a need to find a new material that can replace the materials mentioned above, which must be economical, biocompatible, and biodegradable^26–30^. This work proposed a simple and cost-effective way of utilizing CS waste material for the fabrication of the bio-TENG as shown in Fig. 1a and introduced a promising method for the design of mechanical energy harvesters to provide power to low-power electronic devices.

This study demonstrates a biocompatible and biodegradable (CS-TENG), as shown in Fig. 1b–d. The collagen protein is also one of the most important structural proteins in CS because it gives native tissues strength and elasticity^31^. The biocompatibility of collagen is considered one of its strongest advantages over other synthetic materials^32^. In addition to providing a reliable energy harvester, the proposed approach helps in sustainability by reducing the growing concern worldwide regarding the management of waste generated by one of the largest industries in the world, the poultry and chicken industries. CS contains a significant amount of collagen as electropositive material; it is a good source of inexpensive, abundant, and high-quality feedstock for producing CS-TENGs. CS is a byproduct often overlooked and composed of 55% water, 35% connective tissue, and 5–10% fat^33^. Most of the connective tissues in CS is collagen^34^. Collagen in CS is currently utilized to produce gelatin as a food stabilizer, plasma expander in shock patients, an emulsifying agent, pharmaceutical drug manufacturing and cosmetic products^35^. Collagen is an example of a hierarchical biological material, which provides an interesting example of a fibrous, structural protein with superior mechanical and electrical properties and the basis for the interaction of other tissue components with one another^35^. Therefore, by modulating their structure, collagen modulates the structure and function of tissues^35^. Collagen is capable of providing exceptional mechanical performance because of its ability to organize and interact on a nanometer or micrometer scale. It can be used in a variety of tissue configurations to provide a specialized mechanical performance^36^. It has been known for some time that collagen-rich tissues are piezoelectric, but the role played by collagen piezoelectricity in the body has not been explored^37^. Collagen is a strong structural protein with 29 types, generally consisting of a stable triple helix of two alpha-1 polypeptide chains and one alpha-2 polypeptide chain as shown in Fig. 1e. The chains have a fundamental backbone of amino acids Glycine-X–Y, in which X could either be proline or lysine and Y is the hydroxylated form of either^36^ as shown in Fig. 1e. The building blocks have special properties that give collagen its stable, hydrophilic, and charged nature^36^. Glycine makes about 1/3 of collagen and is an uncharged amino acid. It is also the smallest amino acid due to its side group of 1 hydrogen molecule^36^. This contributes to making the chain firm and tensile. Proline is uncharged, while lysine is positively charged at body pH^38^. Collagen is formed in the rough endoplasmic reticulum (RER) intracellularly and is modified extracellularly. Multiple steps and cofactors are required to form a strong and stable collagen fibril^39^. The first step after the alpha polypeptide chains formed of Glycine and Proline/lysine is hydroxylation of the lysine and proline as shown in Fig. 1e, which requires vitamin C^39^. Next, the hydroxyl-lysine residues are glycosylated with galactose and glucose^39^. This allows the pro-alpha chains to form hydrogen and disulfide bonds resulting in the triple helix structure^39^. This forms procollagen, which will be cleaved from the glycosylated ends extracellularly to form tropocollagen. Crosslinking of the tropocollagen occurs with copper forming the collagen fibril^39^. Tropocollagen molecules measure 280 nm in length and 1.5 nm in diameter, giving them an aspect ratio of 190^40^. It is estimated that tropocollagen molecules are five to ten times stronger than steel^40^ and can easily be bend and twist as shown in Fig. 1b,c. In addition, they can sustain enormous strains of fifty percent or more before fracturing^40^. These intramolecular and intermolecular bonds stabilize the collagen against changes in the body and can only be degraded by special enzymes called collagenases, a form of matrix metalloproteinases (MMPs)^39^. Collagen is a strong and durable structure consisting of a micro-fibril described as a quasi-hexagonal unit cell^35^. Several right-handed twisted micro-fibrils arrange to form a fibril that is found to protect the collagen fibril from degradation from collagenases^40^. This work presents the flexible, eco-friendly, multifunctional CS-TENG, which uses CS film and Kapton film as friction pairs, which are eco-friendly and multifunctional, as shown in Fig. 1b–d. The CS-TENG is both economically and environmentally friendly, as CS film was produced primarily from chicken waste. As a result, the CS-TENG can deliver short-circuit currents of ~ 18 μA, open-circuit voltages of up to ~ 123 V, and output power density of ~ 0.2 mW/cm^2^. CS-TENG can be used to provide a reliable power source for driving low-power devices (light-emitting diodes and stopwatch). The CS-TENG sensor was also utilized as a self-powered sensor to monitor physiological signals within the human body in real-time such as finger touch, and joint movements.

## Results and discussion

### Material characterization

The mechanical strength of CS skin was analyzed using stretching machine as shown in Fig. 1f. Using a stainless-steel razor blade, the CS film sample was initially cut into 20 mm × 10 mm (length × width), after being put on the film tension grippers. At a strain rate of 0.5%/min, uniaxial tensile tests were run with load cell of 20 kN. The CS skin was used to analyze the loading effect and it can hold 17.5 N load under stress condition as shown in Fig. 1f. The surface morphology of CS was analyzed using field emission scanning electron microscope (FESEM) as shown in Fig. 1g and the corresponding color mapping confirms the presence of O-K series and C-K series as shown in Fig. 1h,i. The surface of CS is rough due to collagen fibrous structure, as shown in Fig. 1g, which is also confirmed during the kelvin probe force microscopy (KPFM) analysis of CS.

Figure 1j displays the FTIR spectrum of CS. The N–H stretching vibrations associated with the -NH group of the peptide involved in hydrogen bonds were identified at 3,326 cm^−1^ in the amide band A. The amide band B (30,942 cm^−1^, and 965 cm^−1^) was attributed to the asymmetric stretching of the CH_2_ stretching vibration and the absorption of the CH_2_ alkyl chain. The amide I band at 1658 cm^−1^, the amide II band at1560 cm^−1^, and amide III band at 1253 cm^−1^, originated from C=O stretching, N–H bending vibrations, and C–H stretching, respectively. The presence of a helical structure was indicated by the amide I band, which is associated with the secondary structure of the protein, and the amide III band. These results suggest that helical arrangements exist in the collagen of CS^41–43^.

XRD of CS, as shown in Fig. 1k was analyzed in 2θ range from 10° to 80°. Collagen fibrils contain amorphous components that cause a broad reflection around 2θ ~ 20.05°. From the peak at 2θ ~ 32.1°, we can also determine the axial rise distance between the amino acid residues along the collagen triple helix structure. Collagen's linear translation length is d-0.281 nm in amino acids α-chain. Deconvoluted peaks at 2θ ~ 39.05° and 2θ ~ 42.4° indicate that the N and C telopeptides have axial translation lengths of about 0.228 nm and 0.204 nm, respectively.

Figure 1l,m illustrate the Kelvin probe force microscope (KPFM) technique used to explore the electropositive nature of CS. Because KPFM is a double scan technique by default for ASYLUM MFP-3D AFM, the tip scans over the sample twice during analysis. The topography was created during the first scan as shown in Fig. 1l, and the potential on the surface was analysed during the second scan as shown in Fig. 1m. The electrical tuning of the conducting tip used for the analysis determines the accurate potential on the sample. In this experiment, the optimized potential that produced correct surface potential mapping was 3 V. Figure 3m depicts the topographic character of CS, which has a rms surface roughness of ~ 112.5 nm. The KPFM results of CS show an electropositive potential ranging from + 480 ~  + 568 mV, as shown in Fig. 1m. The KPFM potential scan is plotted in Fig. 1n.

### Device characterization and mechanism

**Figure 2:**
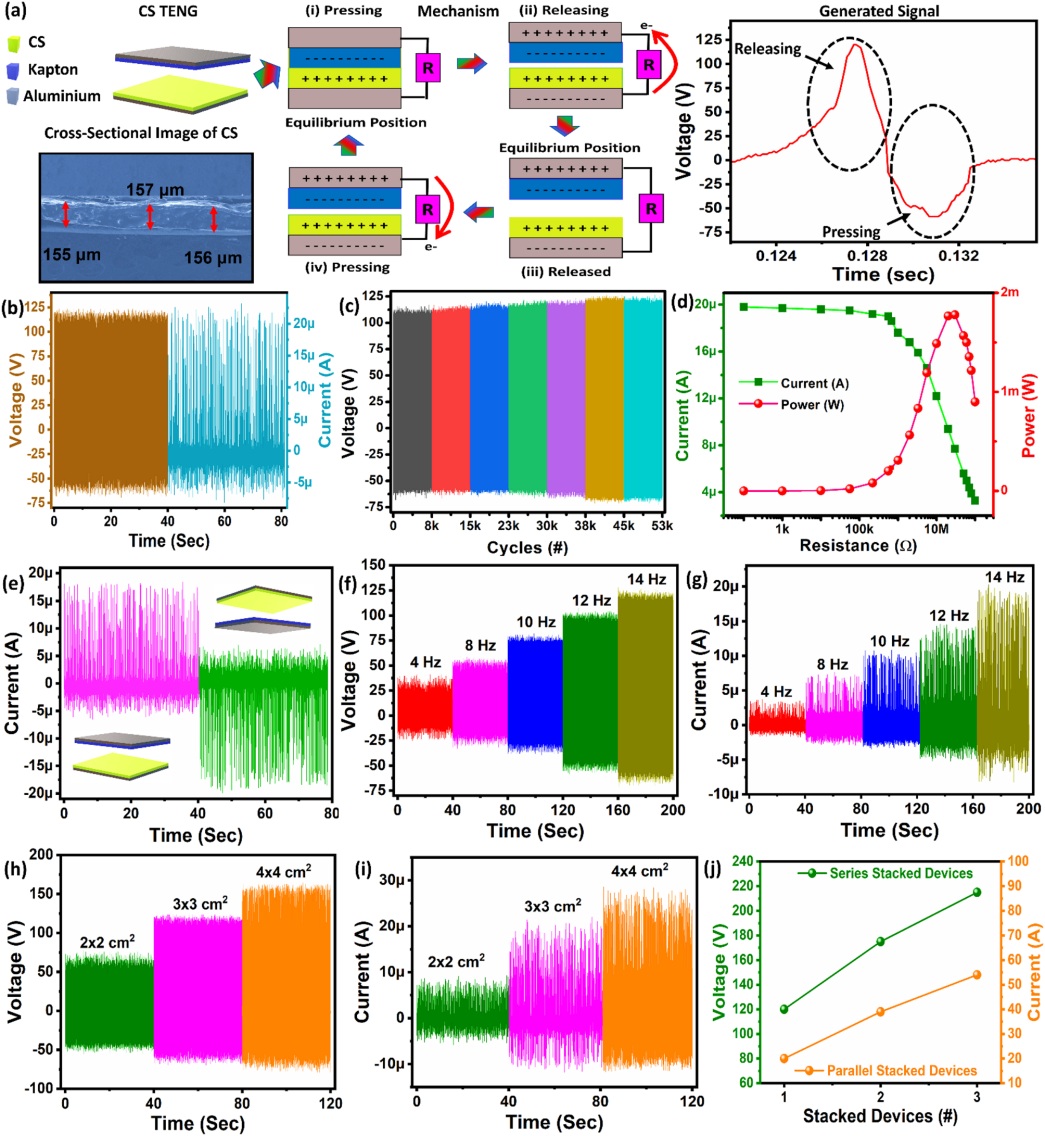
(**a**) Schematic of TENG device, conduction mechanism of CS-TENG and generated signal. (**b**) Open circuit voltage and short circuit current of CS-TENG. (**c**) TENG device stability was analyzed for more than 52,000 cycles. (**d**) Output current and instantaneous power with variable load resistance from 100 Ω to 100 MΩ. (**e**) The phase change response of short circuit current. The frequency response shows an increase of (**f**) open circuit voltage and (**g**) short circuit current by increasing response from 4 Hz, 8 Hz, 10 Hz, 12 Hz, and 14 Hz. The optimization of CS-TENG with different sizes: (**h**) the open circuit voltage and (**i**) the short circuit current of TENG. (**j**) The stacking of CS-TENG in series and parallel.

The TENG was fabricated using Kapton as an electronegative layer and CS as an electropositive layer. In the electrodes, aluminum (Al) conductive adhesive tape was used, and copper wires were affixed to the backside of the electrodes as charge collectors as shown in Fig. 2a. The cross sectional FESEM image of CS is depicting the thickness of ~ 155 µm as shown in Fig. 2a. The dimensions of the TENG device were 2 × 2 cm^2^, 3 × 3 cm^2^, and 4 × 4 cm^2^, and the spacing between the electropositive layer and the electronegative layer was kept as 4 mm. Under constant excitation force, the PET sheet supports the triboelectric dielectric layers for them to press and release quickly. No charge is created or inducted in the initial condition^19^ as shown in Fig. 2a. To establish physical contact between CS (electropositive layer) and Kapton (electronegative layer), an external force must be applied to the device as a result triboelectric charges on the two contacted surfaces will be created^19^, as shown in Fig. 2a(i). The difference between the electron affinities of the two tribo-materials that come into contact with each other. Therefore, the surface of the PET layer is negatively charged, while that of the CS layer is positively charged^19^, as shown in Fig. 2a(i). Then a potential difference can be established once the two contacted surfaces are separated, resulting in an instantaneous electron flow from bottom electrode to top electrode^19^ as shown in Fig. 2a(ii). The electrons continue to flow until the external force is completely withdrawn, at which point the current stops, and eventually achieving balance when the two surfaces are completely apart^19^ as illustrated in Fig. 2a(iii). The electrostatic generated charges will flow back via the external load to compensate for the electric potential difference once the two surfaces are pushed again^19^ as shown in Fig. 2a(iv). The produced current signal during this whole cycle is shown in Fig. 2a.

### Electrical characterization

The device size with dimension of 3 × 3 cm^2^ as shown in supplementary Fig. S1, producing an open circuit voltage (V_oc_) ~ 123 V and short circuit current (I_sc_) ~ 20 µA with operating damping frequency of 14 Hz as shown in Fig. 2b. The characterization setup for CS-TENG is shown in supplementary Fig. S2. It is important for applications of TENGs to have the properties of reliability and stability when they are used in real-life conditions, so the properties of CS-TENGs have to be investigated to ensure their performance for longer time operation. CS-TENG has shown high stability and reliability over 52,000 continuous press-release cycles, as illustrated in Fig. 2c. The SEM image of CS was analyzed after stability test as shown in supplementary Fig. S3. This suggests that CS-TENG is a highly stable and reliable device that can be relied upon without any deterioration in its performance. The output power density was investigated by connecting the device to a variety of resistors that ranged from 1 kΩ to 100 MΩ. With an increase in external loading, the output current of the CS-TENG was observed to decrease, as depicted in Fig. 2d. The following standard Eq. (1) is used to determine output power:

1 $${\text{P}} = {\text{I}}^{{2}} {\text{R}}$$

There are three variables, P, I, and R, representing the output power, the output current,and load resistor through which current is passing. Consequently, the output power density first increased and then decreased with an increase in load resistance, resulting in a decrease in output power density. When the maximum output power of CS-TENG is obtained by applying the maximum power transfer theorem, it can be concluded that the maximum output power can be obtained when the external load resistance equals to the internal impedance of the TENG. It has been found that a maximum peak power of 1.8 mW and power density of 0.2 mW/cm^2^ is achieved at a load resistance of 20 MΩ. It is important to realize that CS-TENGs can deliver a considerable amount of power, which is sufficient for charging wearable electronics, thus greatly extending their operating range. The current phase change test is shown in Fig. 2e, which depicts that the V_oc_ does not contain any external noise. A close relationship exists between the electrical output of the CS-TENG and the operating frequency. The V_oc_ and I_sc_ both increased when the frequency of the contact-release cycle was increased from 4 to 14 Hz (Fig. 2f,g). As can be seen from Eq. (2), I_sc_ increases with the contact-separation speed (v(t)). A faster electron transfer rate at a higher operating frequency is the reason for the increased output current. Equation (3) indicates that the output voltage is independent of the operating frequency; however, the voltage increase is observed here. A possible explanation for this phenomenon could be the fact that, as the operating frequency increases, the force applied to the interface increases, leading to a deformation of the friction layers during contact. Therefore, it is possible that the density (σ) of the triboelectric charges in friction materials will increase due to these factors. Where ε_0_ and ε are the dielectric constants of the air and friction layers, respectively, and d is the thickness of the friction layers (~155 µm) as shown in Fig. 1j. S, σ, d_0_ and x(t) the effective contact area (9 cm^2^), triboelectric charge density, effective thickness, and separation distance, respectively.

2 $${I}_{sc}=\frac{d{Q}_{sc}}{dt}=\frac{S{d}_{0}\sigma }{{\left({d}_{0}+x\left(t\right)\right)}^{2}}\frac{dx}{dt}=\frac{S{d}_{0 }\sigma v\left(t\right)}{\left({d}_{0}+x\left(t\right)\right)}$$

3 $${V}_{0c}=\frac{\sigma x\left(t\right)}{{\varepsilon }_{0}}$$

To illustrate the effect of film size on the performance of CS-TENGs, three different sizes of films were fabricated and characterized, as shown in Fig. 2h,i. For the CS-TENG with an area of 4 × 4 cm^2^, V_0c_ and I_sc_ could reach a maximum of 160 V_oc_ and 28 µA, respectively (Fig. 2h,i). There is a linear relationship between the output performance of the CS-TENG and the area of the friction layers shown in the experiments. To make the experiments feasible in practical applications, the device size 3 × 3 cm^2^ area was selected, resulting in the V_0c_ recorded as 123 V and I_sc_ measured as 20 µA, indicating that the device with the CS-based TENG shows good electrical performance. Single nanogenerator may not be powerful enough for some applications. Various stacking units are linked together in series or parallel to acquire a higher amount of energy. Parallel connection improves driving current whereas series connection raises voltage. As illustrated in Fig. 2j, we demonstrated how to employ stacked CS-TENG devices to enhance the output performance. Here, we measured the I_sc_ and V_oc_ of the different stacking units. The V_oc_ increases as 123 V, 175 V, or 215 V, respectively, when 1, 2, or 3 units are connected in series, as shown in Fig. 2j. In the same way, connecting 1, 2, and 3 units in the parallel circuit depicted in Fig. 2j, respectively, resulted in estimated I_sc_ of 20 μA, 39 μA, and 54 μA as shown in Fig. 2j. The output performance of the CS-TENG may therefore be boosted to the required level by simply creating multiple stacking units, making the device more useful for extensive commercial applications.

### Real life application

**Figure 3:**
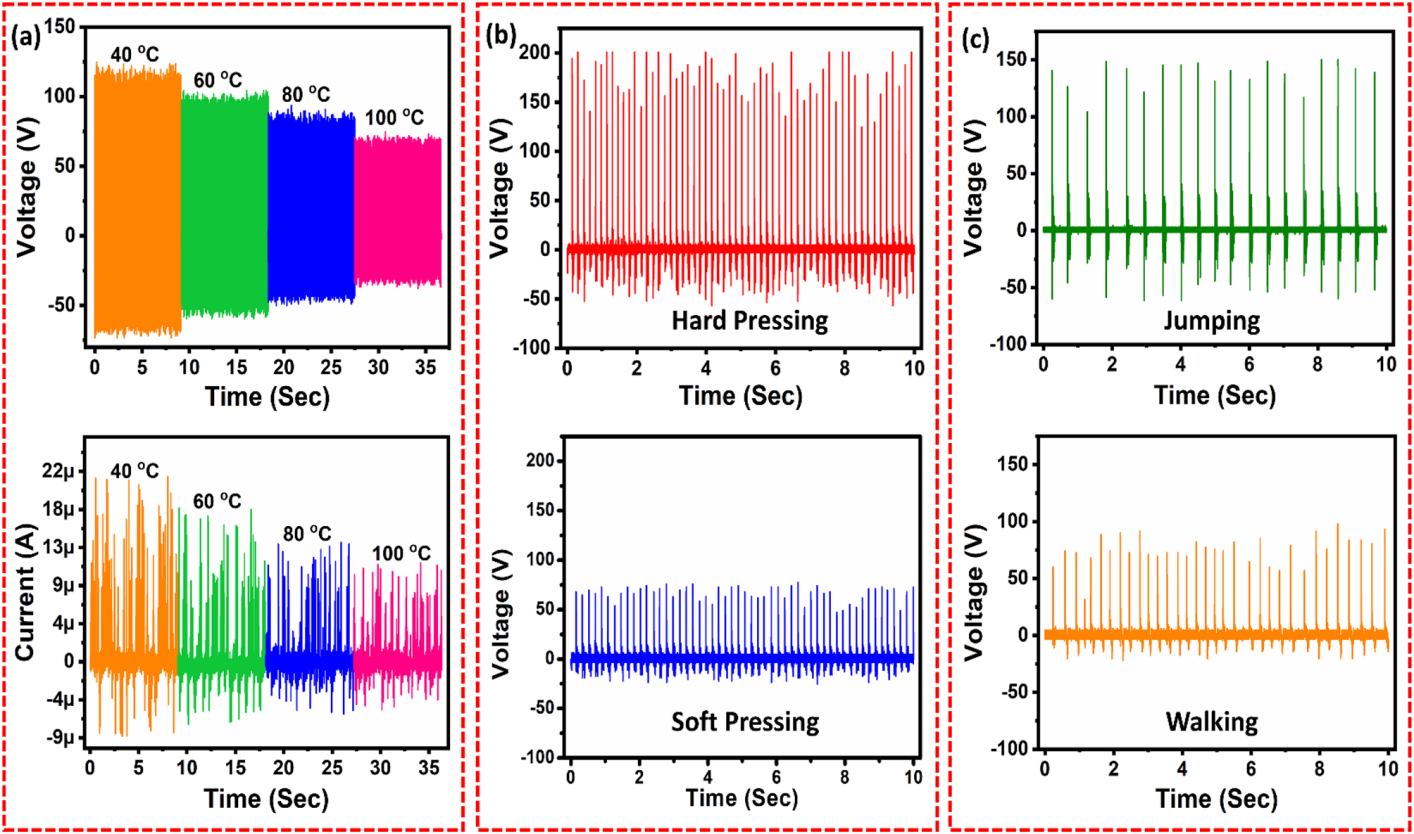
(**a**) Temperature response of the CS-TENG at 40 °C, 60 °C, 80 °C, and 100 °C. Self-powered physiological signal sensing with the CS-TENG: output voltage under (**b**) soft press and hard press, (**c**) jumping and walking.

A further investigation has been conducted to elucidate the V_0c_ and I_sc_ of the CS-TENG with respect to different temperature values, as shown in Fig. 3a. A homemade temperature-controlled linear damping system was used to conduct the temperature test as shown in supplemntry Fig. S2. The results discussed in Fig. 2 were analyzed at room temperature ~ 25 °C. With the increase in the temperature from 40 to 100 °C, the V_0c_ decreases from 123 V to 74 V, and I_sc_ decreases from 20 µA to 11 µA, as shown in Fig. 3a. As a mechanical sensor, the CS-TENG is also suitable for the use of mechanical sensors since it can respond to external forces capacitive or piezoresistive and transfer it into electrical Signals. It should be noted that the CS-TENG is an alternative to traditional mechanical sensors built on principles of capacitive or piezoresistive technologies. The TENGs do not need an external source of power to function, this provides a self-powered approach to detecting physiological signals. As can be seen from Fig. 3b, the CS-TENG can measure the force applied to the device continuously. The hard press generates the V_oc_ of 200 V and soft press generates the V_oc_ 75 V. This CS-TENG can be used for a wide range of applications, including energy harvesting, but it can also be employed to sense mechanical movements of the human body. As a result, CS-TENGs have been mounted on the bottoms of shoes in order to monitor various motion states of the human body, including walking, and jumping. Jumping caused the device to sense a much larger stress, which caused the device to compress much faster, which would explain the large V_0c_ of 150 V. When walking, the device displayed V_oc_ of 98 V as shown in Fig. 3c. The two layers of friction in TENG approach to each other as the foot touches the ground and eventually contact each other to produce a peak voltage when the foot touches the ground. There is an opposite voltage generated when the friction layers separate from each other during the lift up of the foot. Further, compared to walking and running, the positive peak voltage measured in this study was much higher than the negative peak voltage. This may be attributed to the characteristic of jumping, in which CS-TENG is compressed much faster than it is released, which is reflected in the higher ratio of positive to negative voltages. As well as this, there was an oscillation in voltage that might be attributable to the oscillation of shoes in the airborne period, which might contribute to the post-peak oscillation in voltage. The fall-down and standing signal during physiological signal monitoring is shown in supplementary Fig. S4.

**Figure 4:**
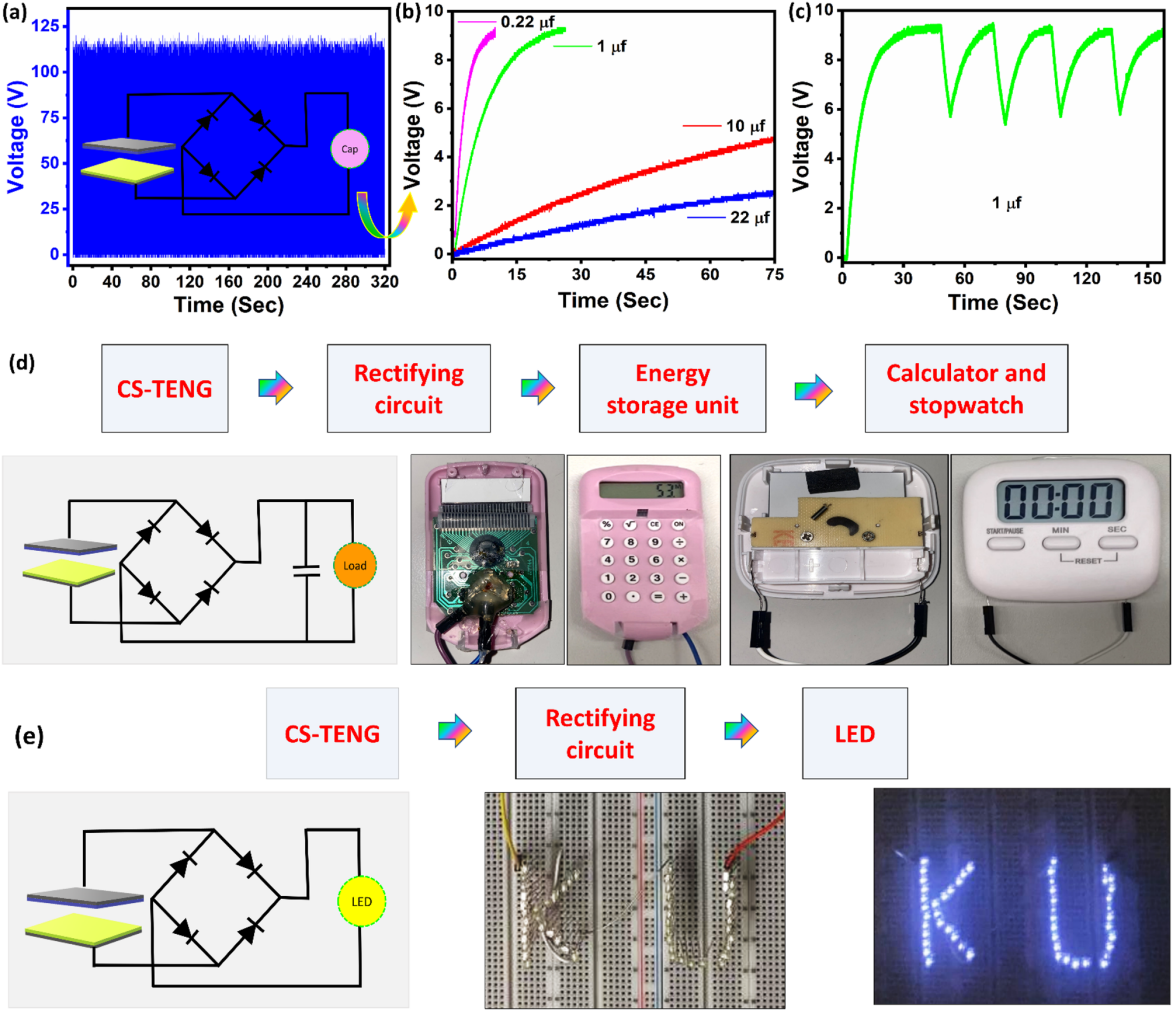
(**a**) The rectified voltage response of the CS-TENG. (**b**) Charging characteristics of the various capacitors (0.22 µF, 1 µF, 10 µF, and 22 µF). (**c**) Charging and discharging of 1 µF capacitor. (**d**) The circuit diagram for the self-driven electronic devices (calculator and stopwatch) using CS-TENG and visual image of the self-driven calculator and stop watch. (**e**) The circuit diagram for LEDs application and the visual image of LEDs lit up using the CS-TENG.

The generated energy from CS-TENG is in the form of an alternating current, which cannot be directly used to power direct-current devices. The interface circuits to the device harvester consist of a rectifier and capacitor. The rectified output voltage performance of the CS-TENG device was used to charge capacitors and drive low-power devices (e.g., LEDs, calculator, etc.) as shown in Fig. 4a. The charging properties of the various capacitors were also assessed over a duration of 75 s up to 9 V, as shown in Fig. 4b. To power the microdevices, the CS-TENG may quickly charge a variety of capacitors in a range of 0.22 µF, 1 µF, 10 µF, and 22 µF as shown in Fig. 4b. The charging and discharging behavior of 1 µF capacitor is analyzed for more than 15 s as shown in Fig. 4c. To demonstrate the overall system performance, the rectified harvested energy stored on the capacitor has been used to power typical consumer electronic devices, in this case, a calculator and stopwatch as shown in Fig. 4d. In addition, the CS-TENG, with a surface area of 9 cm^2^, was able to light up 55 light-emitting diodes (LEDs) connected in series under periodic presses and releases, as shown in Fig. 4e. Based on these results, the CS-TENG can convert biomechanical energy into electric energy.

### Comparison with biomaterials

In this section the performance of CS will be compared with already reported biowaste materials for TENGs. Han et al.^27^, report fish gelatin (FG) based TENG with device structure Cu/PTFE/PDMS/(Space)/FG/Cu. Gelatin was extracted from the fish scale by hydrolysis process. The electropositive and electronegative layer thickness, roughness, and size were optimized to achieve V_oc_ ~ 130 V_oc_ and I_sc_ ~ 0.3 µA and an instantaneous power density of 45.8 µW/cm^2^. Ma et al.^28^, report the fish blade single-electrode TENG as smart electronic skin. The single electrode mode was further investigated as a humidity sensor with a current sensitivity of 446 nAs^2^/m and 50 nA % RH. Further linear accelerator methodology was used for the application of distance sensors. The overall performance of the device achieves V_oc_ ~ 106 V and I_sc_ ~ 7.3 µA and an instantaneous power density of 200 mW/m^2^. Saqib et al.^44^, fabricated eco-friendly nontoxic, and flexible TENG based on peanut shells as biowaste to harvest mechanical energy with device structure (Cu/Peanut Shell/(Space)/PET/Cu. The device operated for more than 6000 cycles. TENG was used as a temperature and humidity sensor for real-life applications. The device operates with V_oc_ ~ 390 V and I_sc_ ~ 14 µA and an instantaneous power density of 0.057 mW/cm^2^. Jayaweera et al.^45^, report TENG based on Human hair. Hair is a highly electropositive material. The hair was processed using Ethanolic NaOH, and the film was fabricated using spin coating with device structure ITO/Kapton/(Spacer)/Hair/ITO. CS-TENG was used for real-time lightning of LEDs. The device operates with V_oc_ ~ 55 V and I_sc_ ~ 5.8 µA and an instantaneous power density of 60 mW/m^2^. This work report CS as the raw material for fabricating the TENG with device structure Al/Kapton/(spacer)/CS/Al. The CS contains a very high amount of collagen with electropositive nature. The performance of TENG was analyzed using different frequency range from 4 Hz to 14 Hz with output performance of V_oc_ ~ 123 V and I_sc_ ~ 20 µA and instantaneous power density of 0.2 mW/cm^2^. The CS-TENG prototype was further utilized as a temperature sensor and self-powered sensor for microelectronic devices and physiological monitoring. The output performance of CS-TENG is comparable with already reported biomaterials based TENGs, as shown in Table 1.

**Table 1:** Comparison of bio-waste-based TENGs with CS-TENG.

No	Bio-waste material	Voltage	Current	Power density	References
1	Fish gelatin	130 V	0.3 µA	45.8 µW/cm^2^	^27^
2	Fish bladder	106 V	7.3 µA	200 mW/m^2^	^28^
3	Peanut shell	390 V	14 µA	0.05 mW/cm^2^	^44^
4	Human hair	55 V	5.8 µA	60 mW/m^2^	^45^
5	Chicken skin	123 V	20 µA	0.2 mW/cm^2^	This Work

## Conclusion

In summary, we have fabricated a flexible, eco-friendly, and multifunctional triboelectric nanogenerator based on CS. Fabricating CS-TENG from Biowaste can convert not only biomechanical energy into electrical energy but also reduces the environmental pollution. CS is a strong electron-donating material and works efficiently as a triboelectric material when paired with the Kapton tape, which possesses a strong electron-accepting ability. The electric output of the CS-TENG with a size of 9 cm^2^ could reach up to 123 V and 20 μA, respectively. The instantaneous power and power density were calculated as 18 mW and 0.2 mW/cm^2^ at a load resistance of 20 MΩ. The outstanding output performance of CS-TENG was demonstrated (for more than 52,000 cycles) as a reliable power source, which could be utilized to charge capacitors, LEDs, electronic calculator, and stopwatch. We have also demonstrated the capability of CS-TENG in sensing physiological signals, showing its high sensitivity to an external force. We believe that the multifunctional CS-TENG, with superior flexibility, low cost, and high performance, will have great potential in the field of portable and wearable electronics.

## Materials and methods

Fresh CS was purchased directly from the local poultry industry to prepare the TENG. In this work, we purchased chicken skin waste from the mart (lulu mart). When chickens are prepared for food, the skin is typically removed and discarded as waste. In this case, utilizing chicken skin for research or study purposes can be considered waste material. Studying chicken skin does not require killing chickens specifically for research purposes. It involves the analysis of a byproduct that is readily available as waste material in the poultry industry, offering opportunities for exploration and potential utilization. The skin of the chicken was thoroughly washed with excessive amounts of water and then let dry in an open atmosphere. On the surface of the skin, any visible fat was mechanically removed. The CS was used as the raw material for fabricating the TENG device Al/Kapton/(spacer)/CS/Al. Mechanical testing on CS film was carried out using a Q800 dynamic mechanical analyzer (TA instruments, USA). To analyze the SEM (Scanning Electron Microscopy) images of CS, a Quanta 250 SEM machine was used with a Schottky field emission gun that provided electrons at 30 kV. Fourier Transform Infrared (FTIR) spectroscopy of CS was performed on a Bruker Alpha system equipped with a diamond attenuated total reflectance (ATR). XRD of CS was investigated using a Bruker D8 diffractometer. Asylum MFP-3D atomic force microscope (AFM) was used for CS Kelvin Probe Force Microscopy (KPFM). The sample's potential was determined using a gold coated Si-tip. The bias is applied to the conducting tip and the sample is grounded for the KFPM procedure. During potential topography, the known bias voltage applied to the tip and generates electrostatic between the tip and sample, which appears as potential on the sample. For imaging, a high-frequency gold coated silicon tip with an apex diameter of 30 nm was employed. The tip resonance frequency is roughly ~ 256 kHz. A linear motor damping system was used to measure the performance of the CS-TENG device by varying the frequency of the motors from 4 to 14 Hz. The linear damping system operates at in a voltage range of 8 V, 12 V, 16 V, 20 V, 24 V and produces frequency in the range of 4 Hz, 8 Hz, 10 Hz, 12 Hz, 14 Hz and produces a resultant force of 1N, 2N, 3N, 4N and 5N. The damping force was measured, using DY220 load cell controller indicator batching display instruments transmitter and DYHW-116 mini button load cell 10 kg compression force sensor. ROHDE&SCHWARZ RTO 1014 oscilloscope 1 GHz 10GSa/S was used to measure open circuit voltage (V_0c_), and KEITHLEY 4200A-SCS was used to measure short circuit current (I_sc_). The dimensions of the CS-TENG device were 4 cm^2^, 9 cm^2^ and 16 cm^2^, and the spacing between the electropositive and electronegative layers was kept at 4 mm.

## Supplementary Information

Supplementary Figures.

## Data Availability

The datasets used and/or analyzed during the current study are available from the corresponding author on reasonable request.
