# Supplementary material for: Chicken skin based Milli Watt range biocompatible triboelectric nanogenerator for biomechanical energy harvesting
Source: Sci Rep. 2023 Jun 22;13:10160. doi: 10.1038/s41598-023-36817-7 (PMC10287749; doi:10.1038/s41598-023-36817-7)
Supplement: Supplementary file 1 — Supplementary Figures. [file 41598_2023_36817_MOESM1_ESM.docx]

**Chicken Skin based Milli Watt range Biocompatible Triboelectric Nanogenerator for Biomechanical Energy Harvesting**

Muhammad Umair Khan^1,2^, Eman Mohammad^3^, Yawar Abbas^2,4^, Moh'd Rezeq^2,4^, Baker Mohammad^1,2,^*

^1^Department of Electrical Engineering and Computer Science, Khalifa University, Abu Dhabi 127788, UAE

^2^System on Chip Lab, Khalifa University, Abu Dhabi 127788, UAE

^3^Sheikh Khalifa Medical City Abu Dhabi, UAE

^4^Department of Physics, Khalifa University, Abu Dhabi 127788, UAE

^*^E-mail: [baker.mohammad@ku.ac.ae](mailto:baker.mohammad@ku.ac.ae)


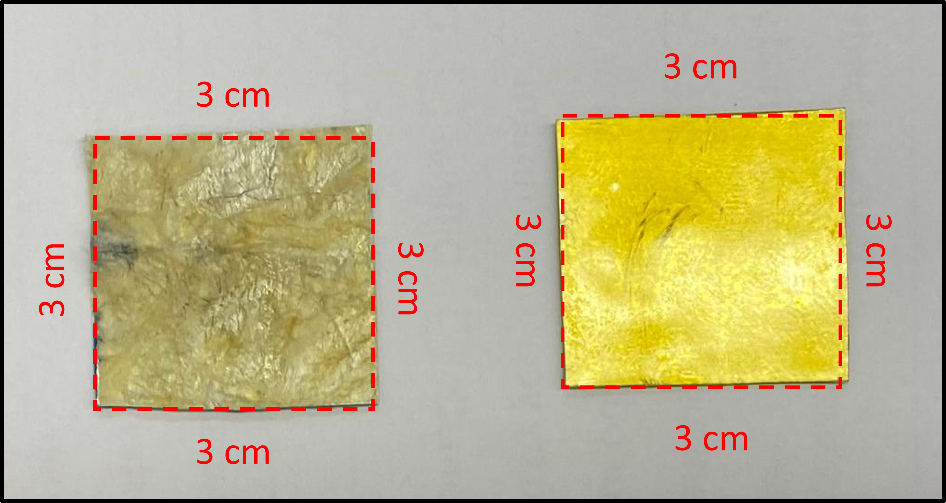


Figure S1. Dimension of CS-TENG.


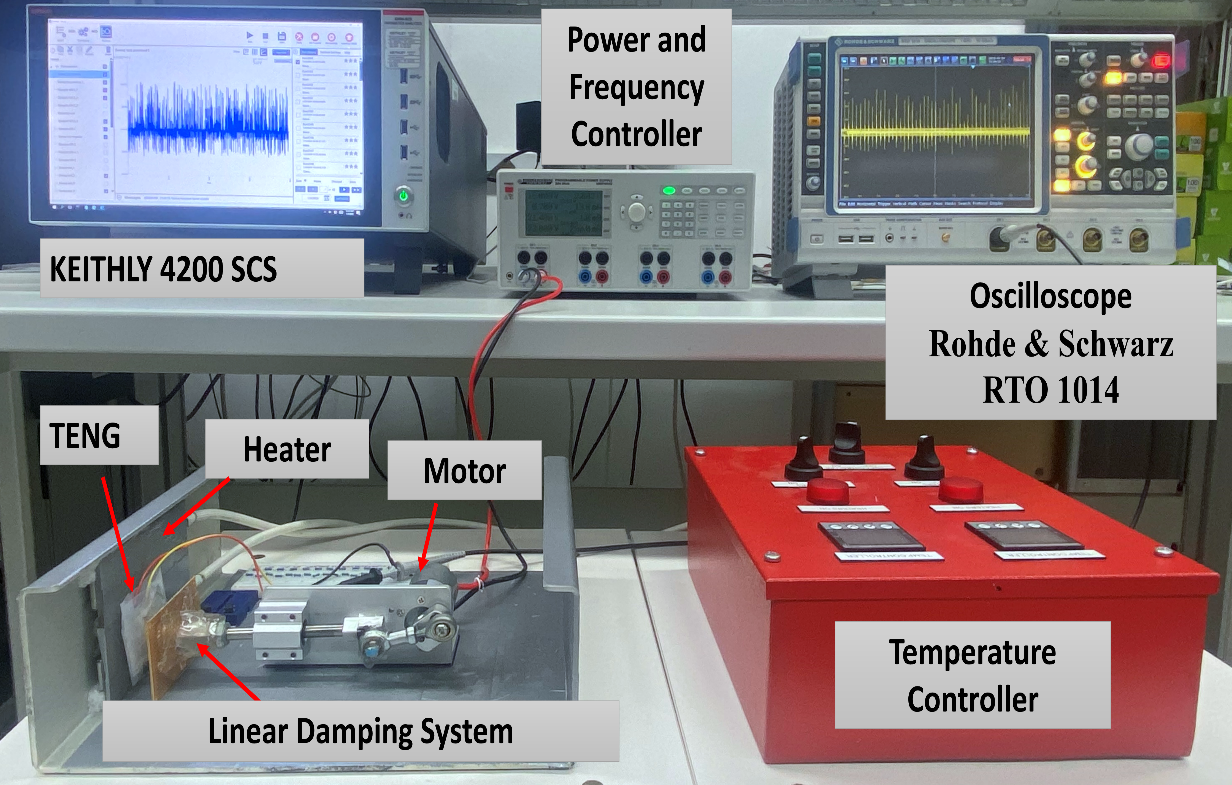


Figure S2. Experiment setup for the characterization of CS-TENG.


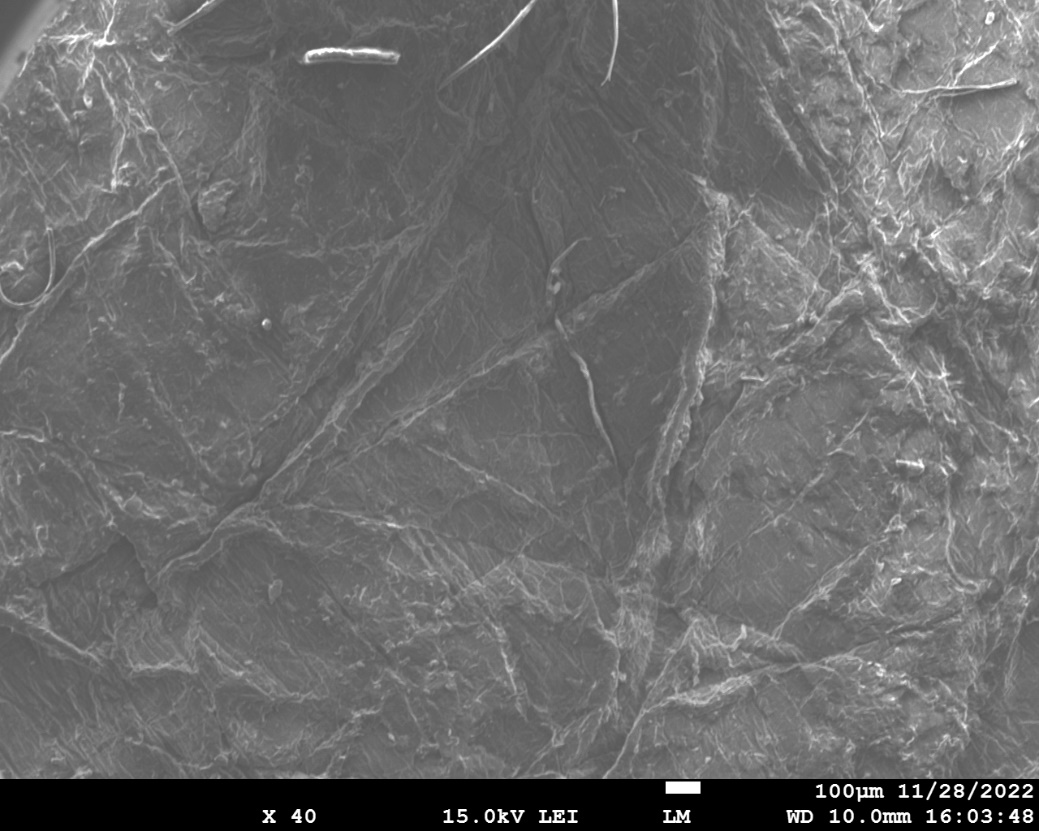


Figure S3. SEM image of CS after Damping.


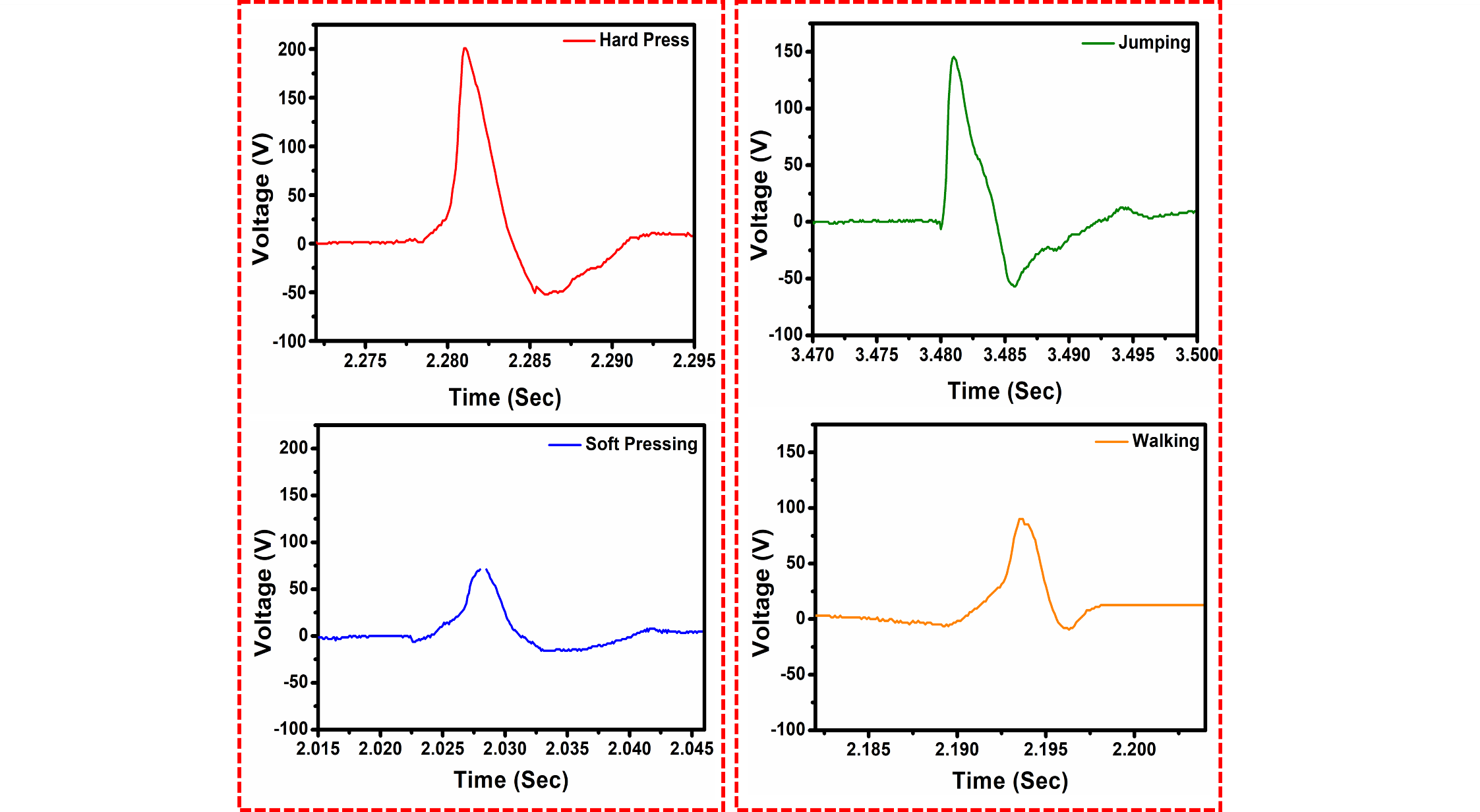


Figure S4. Self-powered physiological zoom signal sensing with the CS-TENG: Output voltage under the soft press, hard press, jumping and walking.
